# Association between adverse pregnancy outcomes and long-term postpartum blood pressure trajectories: a community-based cohort study

**DOI:** 10.3389/fpubh.2026.1924151

**Published:** 2026-10-07

**Authors:** Nan-Di Liu, Li-Hua Chen, Dan-Tong Wang, Yi-Fan Wang, Mei-Chen Yang, Chang-Hao Zu, Hong-Hong He, Hong-Min Liu, Li-Ming Lin, Li-Xia Sun, Xiao-Jia Xu, Hai-Yan Zhao

**Affiliations:** 1 Kailuan General Hospital Affiliated with North China University of Science and Technology, Tangshan, China; 2 North China University of Science and Technology, Tangshan, China; 3 Department of Cardiology, Kailuan General Hospital, Tangshan, China; 4 Hebei North University, Zhangjiakou, China; 5 Kailuan General Hospital, Tangshan, China; 6 Department of Emergency, Affiliated Hospital of North China University of Science and Technology, Tangshan, China; 7 Department of Infection Control, The First Affiliated Hospital of Shantou University Medical College, Shantou, China

**Keywords:** adverse pregnancy outcomes, blood pressure trajectory, community cohort, life-course epidemiology, postpartum hypertension

## Abstract

**Background:**

Adverse pregnancy outcomes (APOs) increase the future risk of hypertension, but their impact on long-term postpartum blood pressure (BP) trajectories remains unclear. We aimed to characterize these trajectories and compare trajectory membership between women with and without APOs.

**Methods:**

This community-based cohort study included 1,346 women with documented deliveries (2004–2020) from the Kailuan Study who underwent at least three postpartum health examinations between 2006 and 2022. APOs included hypertensive disorders of pregnancy (gestational hypertension and preeclampsia/eclampsia), gestational diabetes mellitus, preterm birth, and low birth weight. Generalized additive mixed models were used to characterize longitudinal changes in systolic BP (SBP), diastolic BP (DBP), and mean arterial pressure (MAP). Group-based trajectory modeling was applied to identify distinct BP trajectory groups. Multinomial logistic regression with marginal standardization estimated adjusted risk ratio (aRR).

**Results:**

During a mean follow-up of 9.2 years, SBP showed a nonlinear increase, with an average slope of 1.13 mmHg/year (95% CI, 0.93–1.33) during the first 6.4 years, followed by slower increases of 0.73 and 0.59 mmHg/year during 6.4–10 and 10–15 years, respectively. DBP showed a near-linear increasing pattern, whereas MAP increased linearly. Three trajectory groups were identified for each BP component. APOs were associated with higher risks of high-level SBP (aRR = 1.64; 95% CI: 1.19–2.66), DBP (aRR = 1.61, 95% CI: 1.24–1.87), and MAP (aRR = 2.11, 95% CI: 1.16–3.64) trajectories compared with membership in the low-level BP trajectory groups. Associations varied across APOs subtypes. GDM and PTB were associated with higher risk of membership in the high-level SBP, DBP, and MAP trajectory groups compared with the corresponding low-level trajectory groups.

**Conclusion:**

Different BP components exhibit distinct longitudinal patterns after delivery, APOs are associated with heterogeneous and unfavorable long-term postpartum BP trajectories.

## Introduction

Hypertension is the leading modifiable risk factor for cardiovascular disease worldwide, and its prevalence and impact differ by sex (1, 2). Women experience a steeper blood pressure (BP) increase after age 30 compared with men, suggesting sex specific risk trajectories (3). Pregnancy has been recognized as a natural stress test that reveals a woman’s long-term cardiovascular health (4).

Adverse pregnancy outcomes (APOs), including hypertensive disorders of pregnancy (HDP), gestational diabetes mellitus (GDM), preterm birth (PTB), and low birth weight (LBW), represent a heterogeneous group of pregnancy complications with substantial public health implications (5, 6). The prevalence of individual APOs varies across populations. In China, HDP and GDM affect approximately 7.3 and 14.8% of pregnancies, respectively, whereas PTB and LBW are less frequent (7–10). Despite differences in prevalence, these complications have been increasingly recognized as important markers of future cardiovascular disease, particularly hypertension. For instance, women with HDP have a 6.8-fold higher risk of developing chronic hypertension (11), and GDM is associated with a 59–116% increased risk of hypertension (12). PTB and LBW have also been linked to higher long-term hypertension risk (13, 14).

However, previous studies have primarily relied on single BP measurements or binary hypertension outcomes, which may not adequately capture the dynamic evolution of BP after pregnancy or reveal individual differences in long-term BP patterns. Increasing evidence from longitudinal studies suggests that postpartum BP recovery does not follow a uniform pattern. Particularly among women with HDP, repeated BP assessments have demonstrated substantial heterogeneity in postpartum BP patterns, with some women experiencing persistent BP elevation or delayed BP recovery, whereas others showing more favorable BP trajectories (15, 16). Furthermore, maternal characteristics and pregnancy-related complications may contribute to differences in postpartum BP trajectories, suggesting that pregnancy-related factors could influence long-term BP recovery after pregnancy (17).

Beyond early postpartum BP recovery, recent studies have further emphasized the importance of evaluating long-term cardiovascular risk patterns according to obstetric history. Population-based longitudinal studies have demonstrated that women with a history of APOs may exhibit less favorable trajectories of cardiovascular risk factors, including BP, body weight, and metabolic indicators, and that these differences may persist for years after pregnancy. These findings suggest that APOs may represent not only important clinical events during pregnancy but also early markers of increased long-term cardiovascular risk among women (18). The Kailuan community-based cohort have also investigated the associations of pregnancy-related factors with subsequent cardiovascular risk, including measures of arterial stiffness and other cardiovascular risk factors (19, 20). These studies have provided important evidence regarding the long-term cardiovascular implications of APOs.

Nevertheless, several important knowledge gaps remain. First, previous studies examining postpartum BP trajectories have primarily focused on women with HDP, while the potential influence of other APOs, including GDM, PTB, and LBW, on long-term postpartum BP trajectories remains unclear. Second, although population-based life-course studies have suggested that APOs are associated with unfavorable trajectories of cardiovascular risk factors, including BP, and previous studies from the Kailuan cohort have examined APOs in relation to cardiovascular risk markers, including arterial stiffness, these studies have not specifically characterized longitudinal postpartum BP patterns or identified distinct postpartum BP trajectory groups after pregnancy. Third, limited studies have simultaneously evaluated multiple BP parameters, including systolic BP (SBP), diastolic BP (DBP), and mean arterial pressure (MAP), to comprehensively assess long-term postpartum BP evolution.

In the present study, we focused on four clinically relevant APOs, including HDP(gestational hypertension and preeclampsia/eclampsia), GDM, PTB, and LBW, because these outcomes are relatively common and have been consistently associated with subsequent maternal cardiovascular or metabolic risk (6). These APOs may reflect heterogeneous pregnancy-related vascular, metabolic, and placental abnormalities and may therefore contribute differently to long-term postpartum BP trajectories (21). We aimed to characterize long-term postpartum BP trajectories among women with and without a history of APOs. Additionally, we explored whether associations between individual APOs subtypes and postpartum BP trajectories varied across APOs subtypes.

## Methods

### Study population

We conducted a retrospective cohort analysis using data from the Kailuan prospective cohort study (registration number: ChiCTR-TNRC-11001489). Details have been published previously (20, 22). Data were obtained from previously collected health examination records and historical pregnancy records. Participants were women with documented deliveries between January 2004 and December 2020. The inclusion criteria were: (1) documented delivery during the study period; (2) completion of at least three health examinations at the Kailuan Medical Group between 2006 and 2022 after delivery; and (3) provision of written informed consent for participation in the study. Participants were excluded if fewer than three valid blood pressure measurements were available during follow-up. After applying the inclusion and exclusion criteria, a total of 1,346 participants were included in the final analysis (Figure 1). The study was approved by the ethics committee of Kailuan General Hospital (No. 2006-05). All participants provided written informed consent.

**Figure 1:**
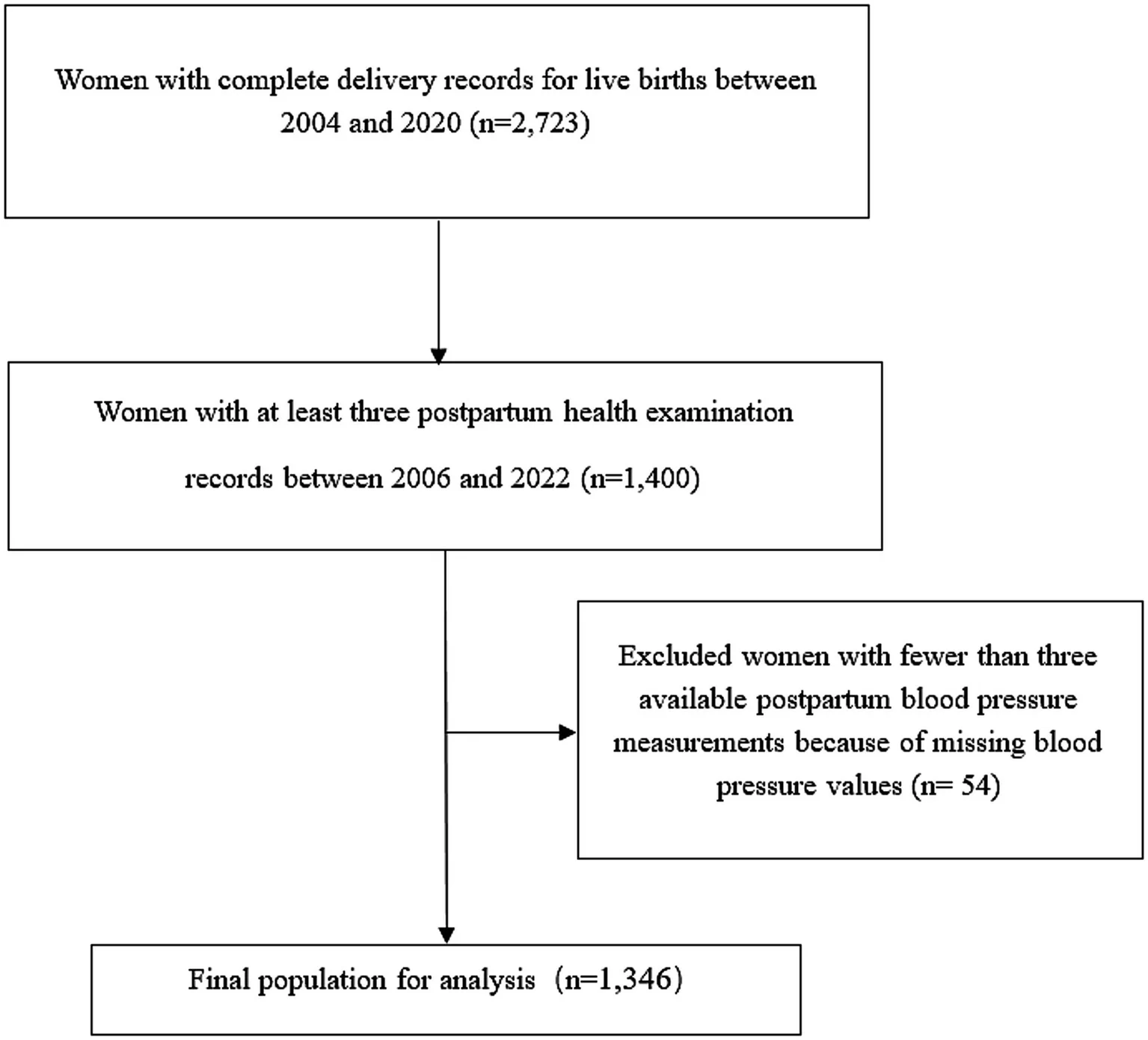
Flowchart for the study population.

### Data collection and definitions

Delivery-related information was systematically collected by trained healthcare professionals using standardized questionnaires and hospitalization medical records. The information included maternal age at delivery, date of delivery, maternal anthropometric measurements (height during pregnancy and weight during pregnancy), blood pressure during pregnancy, neonatal survival status, and maternal and neonatal complications.

Sociodemographic and lifestyle information was collected using standardized questionnaires, including education level, household income, alcohol consumption, and physical activity level.

Information on hypertension status was collected during follow-up visits through self-reported questionnaires, medical records, and physical examinations. Hypertension (23) was defined as the presence of any one of the following: (1) SBP ≥ 140 mmHg and/or DBP ≥ 90 mmHg; (2) current use of antihypertensive medication; or (3) a self-reported physician diagnosis of hypertension.

### Exposure

APOs were identified from hospitalization medical records and standardized questionnaires. The exposure was APOs, defined as the occurrence of one or more of: (1) HDP (24): gestational hypertension (new-onset hypertension after 20 weeks, SBP ≥ 140 mmHg and/or DBP ≥ 90 mmHg, without proteinuria) or preeclampsia/eclampsia (hypertension with proteinuria after 20 weeks); (2) GDM (25): first occurrence during pregnancy of fasting blood glucose (FBG) ≥ 5.1 mmol/L; (3) PTB (26): delivery before 37 weeks of gestation; (4) LBW (26): infant birth weight <2,500 g.

### Follow-up duration and outcomes

The baseline was defined as the date of the first eligible postpartum health examination with BP measurements after delivery. Follow-up duration was defined from this baseline examination after delivery to the last eligible health examination. For women with a subsequent pregnancy, follow-up was censored at the last examination before the subsequent pregnancy; for women without a subsequent pregnancy, follow-up continued until the last available examination.

BP was measured according to standardized procedures of the Kailuan Study, and repeated measurements were averaged for analysis. Mean arterial pressure (MAP) was calculated as DBP + 1/3 × (SBP – DBP) (27). The primary outcome was the long-term dynamic trajectory of postpartum BP. We used GBTM to identify distinct trajectories for SBP, DBP, and MAP, subsequently categorizing them into low-, medium-, and high-level groups based on their level and progression pattern.

### Covariates

The final adjustment set included maternal age at delivery, blood pressure during pregnancy, BMI during pregnancy, household income, education level, physical activity level, and alcohol consumption. Maternal age at delivery, blood pressure during pregnancy, height during pregnancy and weight during pregnancy, were included as continuous variables in the multivariable models. These variables were obtained from medical records or standardized questionnaires. BMI during pregnancy was calculated as weight (kg) during pregnancy divided by height squared (m^2^) during pregnancy. High income was defined as a monthly household income ≥1,000 RMB, higher education was defined as completion of high school or above, and regular physical activity was defined as exercising at least three times per week for at least 30 min per session. Habitual alcohol consumption was defined as current consumption of liquor (>50% alcohol content, >100 mL/day during the past year) or a history of alcohol consumption.

### Statistical analysis

Participants were divided into APOs and Non-APOs groups. Normally distributed continuous data are presented as mean ± standard deviation ( $\bar{x}$ ±s), and compared using the Student’s *t*-test; skewed data as Median (*P*_25_- *P*_75_), with Mann–Whitney *U* test. Categorical data are presented as percentages (%) using the chi-square (*χ*^2^) test.

For women on antihypertensive medication during follow-up (*n* = 67), we imputed BP values by adding 10 mmHg to SBP and 5 mmHg to DBP (28) before fitting all models.

To characterize trends of postpartum BP and compare them between groups, we used generalized additive mixed models (GAMM) with a nonparametric smooth function for time and subject-specific random effects. The fixed-effects part included the main effect of group (APOs vs. Non-APOs) and a smooth term for time fitted separately for each group (time × group interaction). Thin-plate regression splines were used as the smoothing function with 5 basis functions to balance flexibility and computational efficiency. Smoothing parameters were estimated using restricted maximum likelihood (REML). Covariates were selected *a priori* based on previous literature and biological plausibility (Supplementary Figure S1) (16–18, 29–32). Variables considered potential mediators rather than confounders were excluded from the primary adjustment models. Models were adjusted for maternal age at delivery, blood pressure during pregnancy, BMI during pregnancy, household income, education level, physical activity level, and alcohol consumption. Marginal and conditional *R*^2^ values were computed.

To identify latent subgroups with distinct BP developmental trajectories, we applied group-based trajectory modeling (GBTM) to repeated postpartum BP measurements using a censored normal distribution, assuming that missing data were missing at random. GBTM assumes that the population consists of a finite number of latent groups with distinct longitudinal trajectories and allows each group to follow a flexible trajectory shape modeled by polynomial functions, including linear, quadratic, and cubic terms. Therefore, this approach can capture heterogeneous and potentially nonlinear changes in postpartum BP over time.

Model selection followed a stepwise approach. We first determined the optimal polynomial order for each number of trajectory groups by comparing models with different polynomial specifications (cubic, quadratic, linear, and intercept-only terms). The polynomial forms were selected based on model fit indices, including Bayesian Information Criterion (BIC), as well as trajectory interpretability and model parsimony. After determining the appropriate polynomial specification, we sequentially increased the number of trajectory groups from one to two, three, four, or more groups. The optimal number of trajectory groups was determined based on the improvement in Bayesian information criterion (BIC), clinical interpretability, trajectory distinctiveness, and predefined criteria of AvePP >0.7 and a minimum group proportion of 5%. According to these criteria, three trajectory groups (low, medium, and high level) were identified for SBP, DBP, and MAP.

Multinomial logistic regression was used to model membership in the three postpartum BP trajectory groups, with the low-level group as the single reference category. We first examined the association of overall APOs status with BP trajectory-group membership and subsequently repeated the analyses separately for individual APOs subtypes. Multivariable models were adjusted for prespecified potential confounders. The multinomial logistic regression models estimated relative risk ratios (RRRs) for membership in the medium- and high-level trajectory groups compared with the low-level trajectory group. These estimates were subsequently used to obtain model-predicted probabilities. Marginal standardization was applied to these predicted probabilities to estimate the population-averaged probability of membership in each trajectory group under exposed and unexposed conditions, while accounting for the observed distribution of covariates in the study population. Adjusted risk ratios (aRRs) were calculated as the ratio of the standardized marginal probability under the exposed condition to that under the unexposed condition. Ninety-five percent CIs were obtained using 1,000 bootstrap resamples, with the entire regression modeling, prediction, and marginal standardization procedure repeated within each bootstrap sample.

To assess the robustness of our findings, we repeated the analyses after excluding women who used antihypertensive medication during follow-up to minimize the potential influence of antihypertensive treatment on subsequent blood pressure measurements and trajectory patterns.

All analyses were performed using SAS version 9.4 (SAS Institute, Cary, NC, United States) and R version 4.3.2 (R Foundation for Statistical Computing, Vienna, Austria). Two-sided *p* < 0.05 was considered statistically significant.

## Results

### Baseline characteristics

A total of 1,346 women were included in the analysis, with a mean age of 28.5 ± 3.6 years at delivery and 30.2 ± 3.7 years at the first postpartum examination. The mean follow-up duration was 9.2 ± 3.4 years. Among them, 378 (28.1%) women experienced at least one APOs. Compared with women without APOs (*n* = 968), those with APOs were older at delivery and at the first postpartum examination and had higher maternal SBP, DBP, MAP, and BMI levels during pregnancy. The prevalence of hypertension during follow-up was also higher among women with APOs than among those without APOs. Other characteristics, including physical activity, education level, alcohol consumption, and household income, were broadly comparable between the two groups (Table 1). Baseline characteristics across BP trajectory groups are presented in Supplementary Tables S1–S3. A total of 6,628 postpartum BP measurements were available from 1,346 women, with 3–8 measurements per participant (Supplementary Table S4).

**Table 1:** Characteristics of the APOs group and the non-APOs group.

Characteristic	All women	Non-APOs group	APOs group
Baseline examinees, *n*	1,346	968	378
Age at delivery (years), mean ± SD	28.5 ± 3.6	28.4 ± 3.6	28.9 ± 3.7
Age at baseline physical examination (years), mean ± SD	30.2 ± 3.7	30.0 ± 3.7	30.6 ± 3.8
Time from delivery to first examination (years), mean ± SD	1.6 ± 1.1	1.6 ± 1.1	1.7 ± 1.1
Follow-up duration (years), mean ± SD	9.2 ± 3.4	9.2 ± 3.3	9.0 ± 3.4
SBP during pregnancy (mmHg), mean ± SD	118.0 ± 13.7	114.8 ± 10.3	126.3 ± 17.4
DBP during pregnancy (mmHg), mean ± SD	76.4 ± 9.7	74.2 ± 7.2	82.1 ± 12.4
MAP during pregnancy (mmHg), mean ± SD	90.3 ± 10.4	87.7 ± 7.6	96.9 ± 13.4
BMI during pregnancy (kg/m^2^), mean ± SD	28.9 ± 3.5	28.5 ± 3.3	29.7 ± 3.8
Physically active, *n* (%)	51 (3.8)	39 (4.0)	12 (3.2)
Received higher education, *n* (%)	1,049 (77.9)	767 (79.2)	282 (74.6)
Habitual alcohol consumption, *n* (%)	26 (1.9)	16 (1.7)	10 (2.6)
High income, *n* (%)	591 (43.9)	433 (44.7)	158 (41.8)
Hypertension, *n* (%)	19 (1.4)	6 (0.6)	13 (3.4)

### Postpartum blood pressure trajectories according to APOs status (GAMM)

GAMM analyses demonstrated significant longitudinal changes in postpartum SBP, DBP, and MAP over time (all *p* < 0.05). The time trend of SBP showed a clear nonlinear pattern (EDF = 3.437, *p* < 0.001), whereas DBP exhibited a modest deviation from linearity (EDF = 1.220, *p* = 0.005), and MAP followed an approximately linear increasing pattern (EDF = 1.000, *p* < 0.001). The conditional *R*^2^ values were 0.566, 0.516, and 0.577 for SBP, DBP, and MAP, respectively, indicating good model fit (Table 2). Specifically, SBP increased relatively rapidly during the first 6.4 years after delivery, with an average slope of 1.13 mmHg/year (95% CI: 0.93–1.33) over the first 6.4 years. The instantaneous slope reached a maximum of 1.28 mmHg/year (95% CI: 1.08–1.48) at approximately 6.4 years, after which the rate of increase gradually slowed to 0.73 mmHg/year (95% CI: 0.48–0.98) during 6.4–10 years and 0.59 mmHg/year (95% CI: 0.41–0.71) during 10–15 years. In contrast, DBP showed an approximately linear increasing pattern with minimal deviation from linearity, with an average slope of 0.08 mmHg/year (95% CI: 0.02–0.15). MAP increased linearly over the follow-up period, with an average slope of 0.35 mmHg/year (95% CI: 0.30–0.40) (Supplementary Table S5). All three BP components (SBP, DBP, and MAP) showed persistently higher levels among women with APOs compared with those without APOs (Figure 2).

**Table 2:** Fitting results of GAMM for different blood pressure components.

Blood pressure components	EDF	*F*	*p*	Marginal *R*^2^	Conditional *R*^2^
SBP	3.437	170.726	<0.001	0.105	0.566
DBP	1.220	6.730	0.005	0.080	0.516
MAP	1.00	175.117	<0.001	0.111	0.577

The model was adjusted for maternal age at delivery, blood pressure during pregnancy, BMI during pregnancy, household income, education level, physical activity level, and alcohol consumption.

**Figure 2:**
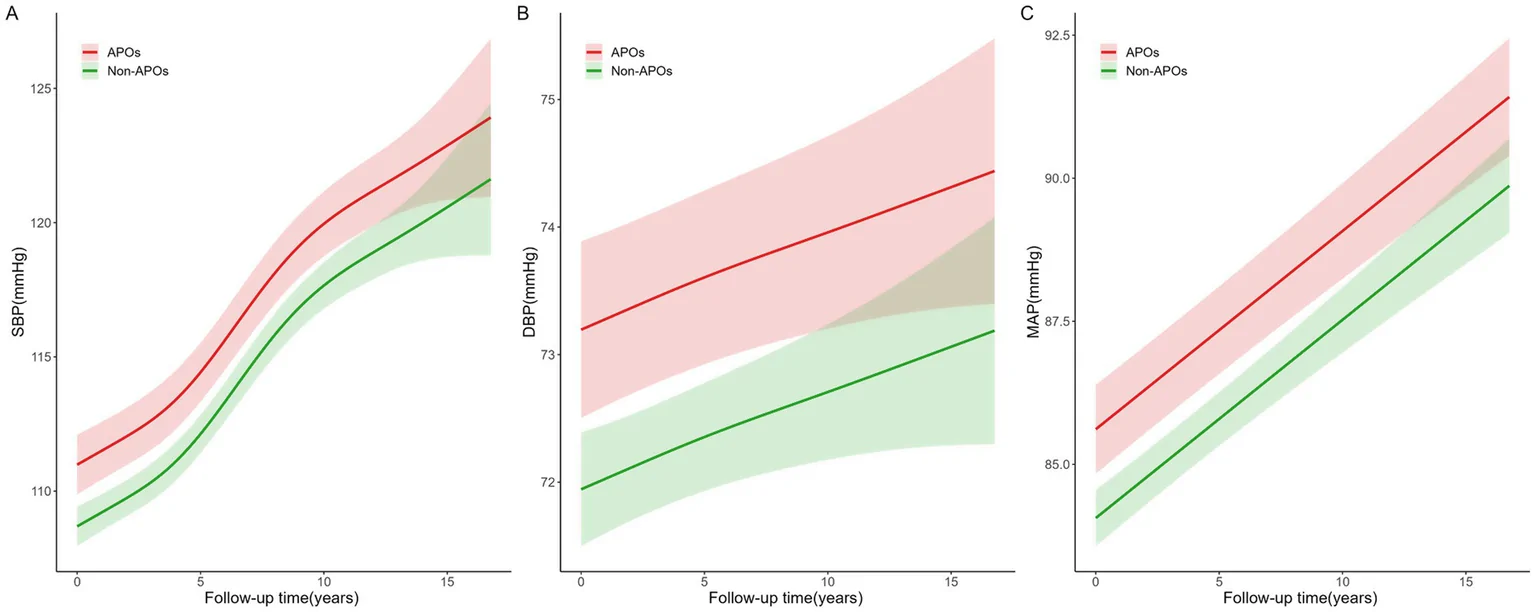
GAMM trajectories of postpartum blood pressure. The graph shows trends in systolic blood pressure **(A)**, diastolic blood pressure **(B)**, and mean arterial pressure **(C)** since the first postpartum exam (years). The x-axis represents the follow-up time, and the y-axis shows the values derived from the generalized additive mixed model. The trends of the adverse and Non-adverse pregnancy outcome groups are represented by the red and green lines, respectively. APOs, adverse pregnancy outcomes; SBP, systolic blood pressure; DBP, diastolic blood pressure; MAP, mean arterial pressure; GAMM, generalized additive mixed model.

### Three distinct BP trajectory groups identified by GBTM

GBTM identified three distinct trajectory groups (low-, medium-, and high-level) for SBP, DBP, and MAP, with the final models selected based on model fit statistics and trajectory interpretability (Supplementary Table S6; Figure 3). The AvePP were all above the recommended threshold of 0.7, indicating good classification accuracy. For SBP, the AvePP values for the low-, medium-, and high-level groups were 0.89, 0.89, and 0.90, respectively; for DBP, the corresponding values were 0.86, 0.86, and 0.86; and for MAP, they were 0.91, 0.89, and 0.91 (Supplementary Tables S7–S9). The proportions of women in the low-, medium-, and high-level trajectory groups were 41.1, 52.1, and 6.8% for SBP; 29.0, 56.1, and 14.9% for DBP; and 46.7, 47.5, and 5.8% for MAP, respectively (Supplementary Tables S10–S12).

**Figure 3:**
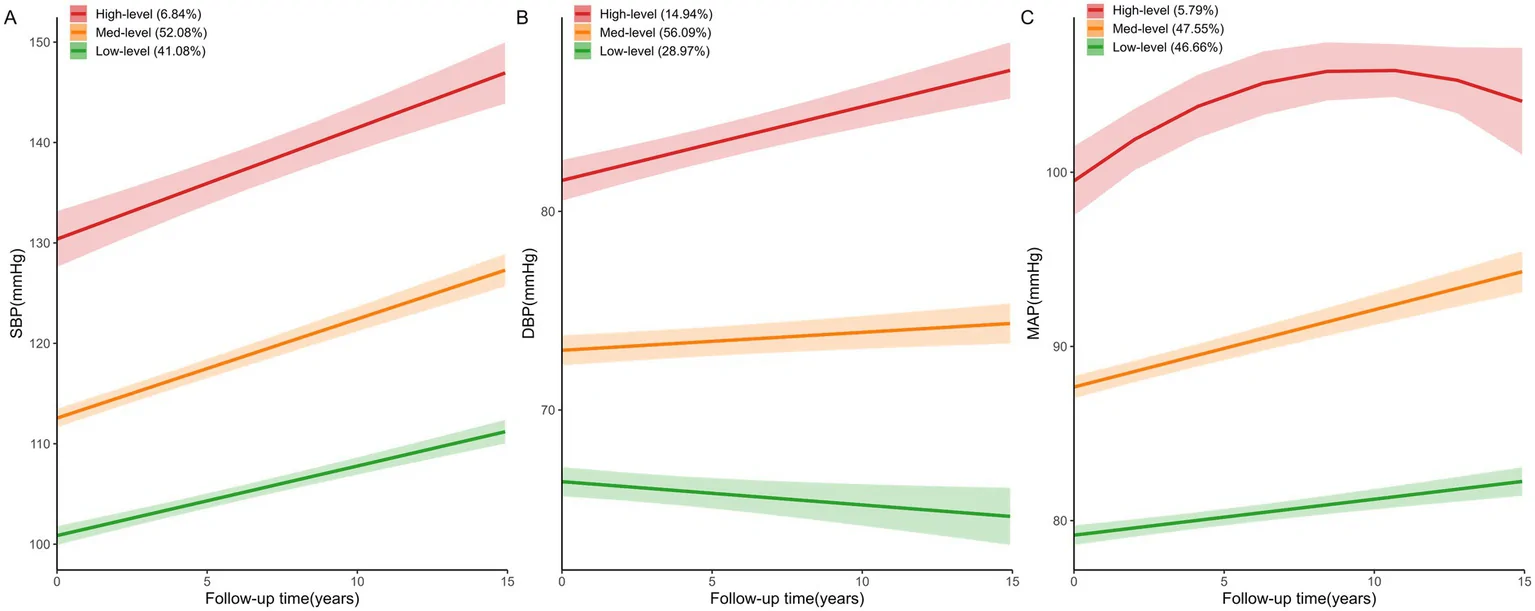
Group-based trajectory modeling of postpartum blood pressure. Multiple trajectories of systolic **(A)**, diastolic **(B)**, and mean arterial pressure **(C)**. The figure shows three trajectories SBP, DBP, and MAP that were identified using group-based trajectory modeling for each component. The solid lines represent the point estimation. SBP, systolic blood pressure; DBP, diastolic blood pressure; MAP, mean arterial pressure.

### Association between APOs and postpartum BP trajectories

Multinomial logistic regression analyses followed by marginal standardization showed that overall APOs status was primarily associated with membership in the high-level postpartum BP trajectory groups (Table 3). Compared with women without APOs, women with APOs had a higher risk of membership in the high-level SBP trajectory group (RR = 2.61, 95% CI: 2.29–4.70) compared with membership in the low-level SBP trajectory group. The association remained significant after adjustment for prespecified confounders, with aRR of 2.49 (95% CI: 2.25–4.33) in Model 2 and 1.64 (95% CI: 1.19–2.66) in Model 3. In contrast, APOs status was not significantly associated with membership in the medium-level SBP trajectory group in either adjusted model.

**Table 3:** Association of APOs with blood pressure trajectory groups.

Blood pressure	Groups	Model 1	Model 2	Model 3
		RR (95%CI)	aRR (95%CI)	aRR (95%CI)
SBP	Low-level trajectory	Ref.	Ref.	Ref.
	Medium-level trajectory	1.12 (0.93–1.25)	1.11 (0.92–1.25)	1.10 (0.92–1.23)
	High-level trajectory	2.61 (2.29–4.70)	2.49 (2.25–4.33)	1.64 (1.19–2.66)
DBP	Low-level trajectory	Ref.	Ref.	Ref.
	Medium-level trajectory	0.97 (0.92–1.06)	0.97 (0.93–1.04)	1.01 (0.94–1.12)
	High-level trajectory	2.16 (1.68–2.45)	2.09 (1.66–2.42)	1.61 (1.24–1.87)
MAP	Low-level trajectory	Ref.	Ref.	Ref.
	Medium-level trajectory	1.12 (0.94–1.22)	1.11 (0.93–1.21)	1.07 (0.91–1.18)
	High-level trajectory	3.28 (2.21–5.66)	3.14 (2.10–5.76)	2.11 (1.16–3.64)

aRR, adjusted risk ratio.

Reference categories: non-APOs (for exposure); low-level trajectory (for outcome).

Model 1: unadjusted; Model 2: adjusted for maternal age at delivery; Model 3: additionally adjusted for blood pressure during pregnancy, BMI during pregnancy, household income, education level, physical activity level, and alcohol consumption based on Model 2.

Similar patterns were observed for DBP and MAP. APOs were associated with a higher risk of membership in the high-level DBP trajectory group (RR = 2.16, 95% CI: 1.68–2.45) compared with membership in the low-level DBP trajectory group, with aRR of 2.09 (95% CI: 1.66–2.42) and 1.61 (95% CI: 1.24–1.87) in Models 2 and 3, respectively. For MAP, APOs were associated with a higher risk of membership in the high-level trajectory group than in the low-level trajectory group (RR = 3.28, 95% CI: 2.21–5.66), with aRR of 3.14 (95% CI: 2.10–5.76) and 2.11 (95% CI: 1.16–3.64) in Models 2 and 3, respectively. There was insufficient evidence for associations between APOs and membership in the medium-level trajectory groups.

### Subtype-specific associations between APOs and postpartum BP trajectories

Associations varied substantially across individual APOs subtypes and BP components (Table 4). Overall, individual APOs subtypes were generally not associated with membership in the medium-level trajectory groups. Associations were observed primarily for the high-level trajectory groups. Compared with membership in the low-level trajectory groups, gestational hypertension was associated with a higher risk of membership in the high-level DBP trajectory group (aRR = 1.99, 95% CI: 1.31–2.35) and the high-level MAP trajectory group (aRR = 1.91, 95% CI: 1.03–3.72), but not the high-level SBP trajectory group (aRR = 1.13, 95% CI: 0.70–1.55).

**Table 4:** Association of APOs subtypes with blood pressure trajectory groups.

APOs	SBP	DBP	MAP
	aRR (95% CI)		
Low-level trajectory	Ref.	Ref.	Ref.
Medium-level trajectory			
Gestational hypertension (*n* = 188)	1.09 (0.96–1.22)	0.96 (0.85–1.12)	1.12 (0.98–1.37)
Preeclampsia/eclampsia (*n* = 55)	0.93 (0.77–1.20)	0.88 (0.65–1.04)	0.87 (0.78–1.13)
GDM (*n* = 171)	1.11 (0.95–1.22)	0.99 (0.88–1.06)	1.04 (0.92–1.19)
PTB (*n* = 76)	1.03 (0.99–1.27)	0.99 (0.91–1.13)	0.90 (0.80–1.11)
LBW (*n* = 44)	1.12 (0.85–1.36)	0.88 (0.60–1.18)	0.86 (0.57–1.14)
High-level trajectory			
Gestational hypertension (*n* = 188)	1.13 (0.70–1.55)	1.99 (1.31–2.35)	1.91 (1.03–3.72)
Preeclampsia/eclampsia (*n* = 55)	1.63 (1.18–2.37)	1.98 (1.37–2.77)	1.60 (0.91–2.76)
GDM (*n* = 171)	2.11 (1.45–3.39)	1.46 (1.03–1.64)	1.74 (1.05–3.26)
PTB (*n* = 76)	2.01 (1.29–2.89)	1.54 (1.24–2.14)	2.56 (1.21–4.08)
LBW (*n* = 44)	1.06 (0.43–2.38)	1.45 (0.66–2.26)	1.28 (0.27–2.88)

aRR, adjusted risk ratio.

Reference categories: non-APOs (for exposure); low-level trajectory (for outcome).

Model 1: unadjusted; Model 2: adjusted for maternal age at delivery; Model 3: additionally adjusted for blood pressure during pregnancy, BMI during pregnancy, household income, education level, physical activity level, and alcohol consumption based on Model 2.

Compared with membership in the corresponding low-level trajectory groups, preeclampsia/eclampsia was associated with a higher risk of membership in the high-level SBP trajectory group (aRR = 1.63, 95% CI: 1.18–2.37) and high-level DBP trajectory group (aRR = 1.98, 95% CI: 1.37–2.77), whereas the association with the high-level MAP trajectory group did not reach statistical significance (aRR = 1.60, 95% CI: 0.91–2.76). Similarly, GDM was associated with a higher risk of membership in the high-level SBP (aRR = 2.11, 95% CI: 1.45–3.39), DBP (aRR = 1.46, 95% CI: 1.03–1.64), and MAP (aRR = 1.74, 95% CI: 1.05–3.26) trajectory groups, compared with the corresponding low-level trajectory groups. PTB was also associated with a higher risk of membership in the high-level SBP (aRR = 2.01, 95% CI: 1.29–2.89), DBP (aRR = 1.54, 95% CI: 1.24–2.14), and MAP (aRR = 2.56, 95% CI: 1.21–4.08) trajectory groups, compared with the corresponding low-level trajectory groups. In contrast, LBW was not significantly associated with membership in any of the high-level trajectory groups compared with the corresponding low-level trajectory groups, with aRR of 1.06 (95% CI: 0.43–2.38), 1.45 (95% CI: 0.66–2.26), and 1.28 (95% CI: 0.27–2.88) for SBP, DBP, and MAP, respectively.

### Sensitivity analyses

Excluding women who used antihypertensive medication during follow-up (*n* = 67) did not materially change the estimates (Supplementary Table S13).

## Discussion

In this large community-based cohort study, we demonstrated that APOs were associated with heterogeneous long-term postpartum BP trajectories. Three findings are noteworthy. First, postpartum BP evolution differed across BP components, with SBP showing a nonlinear pattern, whereas DBP and MAP increased more progressively. Second, APOs were primarily associated with a greater likelihood of belonging to high-level BP trajectory groups compared with low-level trajectory groups. Third, these associations varied substantially by APOs subtype, suggesting that pregnancy complications may serve as indicators associated with future cardiovascular vulnerability.

Previous studies have consistently demonstrated that APOs, particularly HDP and GDM, are associated with increased risks of subsequent hypertension and cardiovascular disease (33–35). More recently, emerging studies have begun to characterize longitudinal postpartum BP patterns among women with HDP. Hauspurg et al. reported distinct postpartum ambulatory BP patterns following new-onset HDP, highlighting persistent BP elevation beyond the immediate postpartum period (15). Other studies further demonstrated heterogeneous postpartum BP trajectories among women after HDP, with variations according to racial background and baseline metabolic risk profiles (16, 17). In addition, life-course analyses have suggested that obstetric history may influence the long-term trajectory of maternal cardiovascular risk factors (18). Evidence from the Kailuan community-based cohort also supports the potential long-term cardiovascular consequences of APOs. Liu et al. reported that women with APOs had a higher prevalence of vascular aging, as assessed by brachial-ankle pulse wave velocity, than women without APOs (19). Similarly, a recent study from the Kailuan cohort found that pregnancy-induced hypertension was associated with a higher estimated lifetime risk of atherosclerotic cardiovascular disease (20). Despite these findings, important gaps remain. Existing longitudinal trajectory studies have largely focused on women with HDP, while evidence regarding the broader spectrum of APOs and their associations with long-term postpartum BP trajectories remains limited. Moreover, previous studies have primarily assessed postpartum BP at discrete time points or focused on specific cardiovascular phenotypes, such as vascular aging or estimated cardiovascular risk. In contrast, our study examined a broader spectrum of APOs and characterized the longitudinal evolution of SBP, DBP, and MAP using trajectory-based approaches.

The distinct temporal patterns observed across BP components provide additional insight into the long-term evolution of BP after pregnancy. The nonlinear pattern observed for SBP and the relatively linear increases in DBP and MAP provide further insights into the dynamic nature of postpartum BP changes. The initial rapid increase followed by gradual deceleration in SBP may reflect progressive vascular aging and accumulation of cardiovascular risk factors, as arterial stiffness increases with age and contributes to long-term SBP elevation (36, 37). In contrast, the persistent upward trends in DBP and MAP may indicate sustained alterations in vascular resistance and overall hemodynamic load, consistent with previous longitudinal studies demonstrating distinct trajectories of different BP components across the life course (38). These findings underscore the importance of evaluating BP as a longitudinal phenotype rather than relying solely on isolated measurements, as individuals with comparable BP levels at a single time point may subsequently follow different BP trajectories and cardiovascular risk profiles (39).

Several biological mechanisms may underlie the associations between APOs and unfavorable postpartum BP trajectories. Pregnancy complications may reflect underlying vascular, metabolic, and inflammatory susceptibility that persists beyond pregnancy. HDP, including gestational hypertension and preeclampsia, have been associated with persistent vascular dysfunction after delivery, including impaired endothelial function and increased arterial stiffness, which may contribute to long-term BP elevation (40). Notably, women with a history of preeclampsia have been shown to exhibit persistent microvascular dysfunction 15–25 years after pregnancy, while women with a history of hypertensive pregnancy have also been reported to have greater left ventricular mass more than 25 years after pregnancy (41, 42). Similarly, GDM may reflect persistent metabolic dysfunction and is associated with increased long-term cardiometabolic risk, including subsequent cardiovascular disease and metabolic disorders (43). PTB may also serve as a marker of underlying maternal vascular, metabolic, or placental dysfunction and has been associated with increased long-term maternal cardiovascular risk (44, 45). These distinct pathophysiological pathways may partly explain the heterogeneous associations observed across APOs subtypes and BP components.

The strengths of this study include its community-based cohort design, systematic longitudinal BP measurements, comprehensive evaluation of multiple APOs subtypes, and application of advanced statistical approaches to characterize both temporal BP patterns and trajectory heterogeneity. Several limitations should be acknowledged. First, although data were derived from the Kailuan Study, this analysis was conducted retrospectively within a prospective cohort using previously collected health examination data and historical pregnancy records. Therefore, potential misclassification of pregnancy-related exposures cannot be completely excluded. Second, because BP measurements were not available immediately after delivery, and the first postpartum BP assessment occurred on average 1.6 years after childbirth, we were unable to evaluate BP changes during the early postpartum period. Thus, our findings primarily reflect sustained long-term associations rather than immediate postpartum BP responses. Third, participants were recruited from a single industrial cohort in northern China, which may limit generalizability to populations with different geographic, socioeconomic, or healthcare characteristics. Fourth, trajectory groups were empirically derived from the observed data and do not directly correspond to established clinical BP categories. Fifth, APOs were analyzed as a composite exposure despite the heterogeneous and potentially interrelated nature of individual APO subtypes. For example, HDP may increase the risk of PTB. Thus, treating APOs as a single exposure may not fully capture the distinct relationships among individual APOs or their potentially heterogeneous associations with postpartum BP trajectories. Although we examined individual APOs subtypes separately, the potential interrelationships among these outcomes cannot be completely accounted for.

## Conclusion

In conclusion, this study demonstrates that APOs are associated with unfavorable long-term postpartum BP trajectories. Different BP components showed distinct temporal patterns after pregnancy, with SBP exhibiting a nonlinear trajectory and DBP and MAP showing progressive increases over time. Women with APOs had higher risks of belonging to high-level BP trajectory groups compared with low-level trajectory groups, although the magnitude and pattern of associations varied by APOs subtype. These findings emphasize the importance of long-term, individualized BP surveillance after pregnancy and may support the integration of pregnancy complications into cardiovascular risk assessment and prevention strategies.

## Data Availability

The raw data supporting the conclusions of this article will be made available by the authors, without undue reservation.
