# Supplementary material for: Association between adverse pregnancy outcomes and long-term postpartum blood pressure trajectories: a community-based cohort study
Source: Front Public Health. 2026 Oct 7;14:1924151. doi: 10.3389/fpubh.2026.1924151 (PMC13683097; doi:10.3389/fpubh.2026.1924151)
Supplement: Supplementary file 1 [file Table_1.DOCX]

**Supplementary Materials**

[**Table S1.** Comparison of Baseline Characteristics Among SBP Trajectory Groups	2](#_Toc239000770)

[**Table S2.** Comparison of Baseline Characteristics Among DBP Trajectory Groups	3](#_Toc239000771)

[**Table S3.** Comparison of Baseline Characteristics Among MAP Trajectory Groups	4](#_Toc239000772)

[**Table S4.** Distribution of repeated blood pressure measurements among women included in trajectory analyses (n=1,346)	5](#_Toc239000773)

[**Table S5.** Estimated average annual slopes of postpartum BP trajectories during different follow-up intervals (0–15 years after delivery)	5](#_Toc239000774)

[**Table S6.** Model selection for blood pressure trajectory models with different polynomial specifications	6](#_Toc239000775)

[**Table S7.** Evaluation metrics for the goodness-of-fit of different SBP trajectory models	6](#_Toc239000776)

[**Table S8.** Evaluation metrics for the goodness-of-fit of different DBP trajectory models	7](#_Toc239000777)

[**Table S9.** Evaluation metrics for the goodness-of-fit of different MAP trajectory models	7](#_Toc239000778)

[**Table S10.** Parameter estimates for polynomial terms of SBP trajectory models	8](#_Toc239000779)

[**Table S11.** Parameter estimates for polynomial terms of DBP trajectory models	8](#_Toc239000780)

[**Table S12.** Parameter estimates for polynomial terms of MAP trajectory models	9](#_Toc239000781)

[**Table S13.** Sensitivity analysis excluding individuals who developed hypertension during follow-up (N=67)	9](#_Toc239000782)

[**Figure S1.** Directed acyclic graph (DAG)	10](#_Toc239000783)

# **Table S1.** Comparison of Baseline Characteristics Among SBP Trajectory Groups

| Characteristic | All women | Low-level | Medium-level | High-level |
| --- | --- | --- | --- | --- |
| Baseline examinees, n | 1,346 | 553 | 701 | 92 |
| Age at delivery (years), mean ± SD | 28.5±3.6 | 27.9±3.1 | 28.7±3.6 | 30.5±5.3 |
| Age at baseline physical examination (years), mean ± SD | 30.2±3.7 | 29.5±3.3 | 30.4±3.6 | 32.2±5.5 |
| Time from delivery to first examination (years), mean ± SD | 1.6±1.1 | 1.6±1.1 | 1.7±1.1 | 1.7±1.4 |
| Follow-up duration (years), mean ± SD | 9.2±3.4 | 10.0±3.1 | 8.6±3.4 | 8.6±3.6 |
| Prepregnancy SBP (mmHg), mean ± SD | 118.0±13.7 | 116.2±13.2 | 118.3±12.7 | 126.7±19.3 |
| Prepregnancy DBP (mmHg), mean ± SD | 76.4±9.7 | 75.2±8.8 | 76.7±9.5 | 81.6±13.3 |
| Prepregnancy MAP (mmHg), mean ± SD | 90.3±10.4 | 88.8±9.6 | 90.6±10.1 | 96.7±14.9 |
| Prepregnancy BMI (kg/m^2^), mean ± SD | 28.9±3.5 | 28.0±3.2 | 29.4±3.5 | 30.4±4.2 |
| Physically active, n (%) | 51 (3.8) | 19 (3.4) | 28 (4.0) | 4 (4.3) |
| Received higher education, n (%) | 1049 (77.9) | 441 (79.7) | 542 (77.3) | 66 (71.7) |
| High income, n (%) | 591 (43.9) | 241 (43.6) | 315 (44.9) | 35 (38.0) |
| Habitual alcohol consumption, n (%) | 26 (1.9) | 10 (1.8) | 14 (2.0) | 2 (2.2) |
| Hypertension, n (%) | 19 (1.4) | 1 (0.2) | 4 (0.6) | 14 (15.2) |
| APOs, n (%) | 378 (28.1) | 118 (21.3) | 213 (30.4) | 47 (51.1) |

# **Table S2.** Comparison of Baseline Characteristics Among DBP Trajectory Groups

| Characteristic | All women | Low-level | Medium-level | High-level |
| --- | --- | --- | --- | --- |
| Baseline examinees, n | 1,346 | 390 | 755 | 201 |
| Age at delivery (years), mean ± SD | 28.5±3.6 | 27.8±3.1 | 28.7±3.6 | 29.2±4.3 |
| Age at baseline physical examination (years), mean ± SD | 30.2±3.7 | 29.4±3.2 | 30.3±3.7 | 31.1±4.4 |
| Time from delivery to first examination (years), mean ± SD | 1.6±1.1 | 1.5±1.0 | 1.6±1.1 | 1.9±1.3 |
| Follow-up duration (years), mean ± SD | 9.2±3.4 | 9.2±3.3 | 9.2±3.3 | 9.3±3.6 |
| Prepregnancy SBP (mmHg), mean ± SD | 118.0±13.7 | 115.8±12.0 | 117.7±13.4 | 123.7±16.3 |
| Prepregnancy DBP (mmHg), mean ± SD | 76.4±9.7 | 74.9±8.9 | 76.1±9.1 | 80.2±11.9 |
| Prepregnancy MAP (mmHg), mean ± SD | 90.3±10.4 | 88.6±9.3 | 90.0±9.9 | 94.7±12.9 |
| Prepregnancy BMI (kg/m^2^), mean ± SD | 28.9±3.5 | 28.0±3.1 | 28.9±3.5 | 30.4±3.9 |
| Physically active, n (%) | 51 (3.8) | 13 (3.3) | 28 (3.7) | 10 (5.0) |
| Received higher education, n (%) | 1049 (77.9) | 311 (79.7) | 598 (79.2) | 140 (69.7) |
| High income, n (%) | 591 (43.9) | 181 (46.4) | 327 (43.3) | 83 (41.3) |
| Habitual alcohol consumption, n (%) | 26 (1.9) | 10 (2.6) | 12 (1.6) | 4 (2.0) |
| Hypertension, n (%) | 19 (1.4) | 1 (0.3) | 3 (0.4) | 15 (7.5) |
| APOs, n (%) | 378 (28.1) | 76 (19.5) | 212 (28.1) | 90 (44.8) |

# **Table S3.** Comparison of Baseline Characteristics Among MAP Trajectory Groups

| Characteristic | All women | Low-level | Medium-level | High-level |
| --- | --- | --- | --- | --- |
| Baseline examinees, n | 1,346 | 628 | 640 | 78 |
| Age at delivery (years), mean ± SD | 28.5±3.6 | 28.0±3.2 | 28.8±3.7 | 30.1±5.0 |
| Age at baseline physical examination (years), mean ± SD | 30.2±3.7 | 29.6±3.3 | 30.5±3.8 | 31.9±5.2 |
| Time from delivery to first examination (years), mean ± SD | 1.6±1.1 | 1.6±1.0 | 1.7±1.2 | 1.8±1.4 |
| Follow-up duration (years), mean ± SD | 9.2±3.4 | 9.5±3.3 | 8.8±3.4 | 9.8±3.6 |
| Prepregnancy SBP (mmHg), mean ± SD | 118.0±13.7 | 116.1±13.0 | 118.9±13.2 | 127.1±18.1 |
| Prepregnancy DBP (mmHg), mean ± SD | 76.4±9.7 | 75.1±8.9 | 76.9±9.6 | 82.8±12.9 |
| Prepregnancy MAP (mmHg), mean ± SD | 90.3±10.4 | 88.7±9.6 | 90.9±10.3 | 97.6±14.1 |
| Prepregnancy BMI (kg/m^2^), mean ± SD | 28.9±3.5 | 28.1±3.2 | 29.3±3.5 | 31.0±4.4 |
| Physically active, n (%) | 51 (3.8) | 19 (3.0) | 30 (4.7) | 2 (2.6) |
| Received higher education, n (%) | 1049 (77.9) | 500 (79.6) | 502 (78.4) | 47 (60.3) |
| High income, n (%) | 591 (43.9) | 276 (43.9) | 284 (44.4) | 31 (39.7) |
| Habitual alcohol consumption, n (%) | 26 (1.9) | 12 (1.9) | 13 (2.0) | 1 (1.3) |
| Hypertension, n (%) | 19 (1.4) | 2 (0.3) | 5 (0.8) | 12 (15.4) |
| APOs, n (%) | 378 (28.1) | 139 (22.1) | 196 (30.6) | 43 (55.1) |

# **Table S4.** Distribution of repeated blood pressure measurements among women included in trajectory analyses (n=1,346)

| Number of measurements | Frequency (n) | Percent (%) |
| --- | --- | --- |
| 3 | 308 | 22.88% |
| 4 | 284 | 21.10% |
| 5 | 245 | 18.20% |
| 6 | 263 | 19.54% |
| 7 | 203 | 15.08% |
| 8 | 43 | 3.20% |

# **Table S5.** Estimated average annual slopes of postpartum BP trajectories during different follow-up intervals (0–15 years after delivery)

| BP component | Period, years | Estimated slope, mmHg/year | 95% CI |
| --- | --- | --- | --- |
| SBP | 0-6.4 | 1.13 | (0.93-1.33) |
|  | 6.4-10 | 0.73 | (0.48-0.98) |
|  | 10-15 | 0.59 | (0.41-0.71) |
| DBP | 0-15 | 0.08 | (0.02-0.15) |
| MAP | 0-15 | 0.35 | (0.30-0.40) |

For SBP, the instantaneous slope reached a maximum of 1.28 mmHg/year (95% CI, 1.08–1.48) at approximately 6.4 years. Slopes for specified intervals represent average annual changes over each period. DBP showed only a minor deviation from linearity (EDF=1.220), and was therefore considered approximately linear.

# **Table S6.** Model selection for blood pressure trajectory models with different polynomial specifications

|  | SBP | | DBP | | MAP | |
| --- | --- | --- | --- | --- | --- | --- |
|  | BIC | AIC | BIC | AIC | BIC | AIC |
| Cubic/ Cubic/ Cubic | -25496.64 | -25457.60 | -22835.70 | -22796.66 | -23226.14 | -23187.10 |
| Quadratic/ Quadratic / Quadratic | -26493.87 | -25462.64 | -22826.55 | -22795.32 | -23216.11 | -23184.88 |
| Linear/ Linear/ Linear | -25486.54 | -25452.47 | -22811.37 | -22793.65 | -23210.84 | -23187.42 |

# **Table S7.** Evaluation metrics for the goodness-of-fit of different SBP trajectory models

| Group counts | Avepp (%) | Proportions per class (%) | BIC | △BIC |
| --- | --- | --- | --- | --- |
| 1 | 100 | 100 | -26545.87 | 3.98 |
| 2 | 92.96-89.36 | 62.78-37.22 | -25781.45 | 7.97 |
| 3 | 89.99-88.75-89.85 | 40.56-52.67-6.77 | -25496.64 | 12.00 |
| 4 | 88.85-86.27-86.71-97.27 | 27.93-54.23-16.12-1.72 | -25352.07 | 15.94 |

# **Table S8.** Evaluation metrics for the goodness-of-fit of different DBP trajectory models

| Group counts | Avepp (%) | Proportions per class (%) | BIC | △BIC |
| --- | --- | --- | --- | --- |
| 1 | 100 | 100 | -23759.75 | 3.99 |
| 2 | 93.61-90.23 | 69.69-30.31 | -23043.59 | 7.97 |
| 3 | 86.37-85.64-86.01 | 29.42-56.39-14.19 | -22835.70 | 11.96 |
| 4 | 83.85-85.53-86.43-88.41 | 19.17-56.24-22.21-2.38 | -22711.72 | 15.95 |

# **Table S9.** Evaluation metrics for the goodness-of-fit of different MAP trajectory models

| Group counts | Avepp (%) | Proportions per class (%) | BIC | △BIC |
| --- | --- | --- | --- | --- |
| 1 | 100 | 100 | -24348.33 | 3.99 |
| 2 | 93.68-90.47 | 67.31-32.69 | -23511.97 | 7.97 |
| 3 | 90.65-89.40-90.79 | 46.58-47.47-5.95 | -23226.14 | 11.96 |
| 4 | 87.21-85.67-87.30-98.05 | 24.74-52.53-21.17-1.56 | -23067.08 | 15.94 |

# **Table S10.** Parameter estimates for polynomial terms of SBP trajectory models

| Group | Trajectory groups | Polynomial form of model | Estimate | SE | *t* | *P* |
| --- | --- | --- | --- | --- | --- | --- |
| 3 | Low-level (n=553) | Intercept | 100.845 | 0.444 | 227.371 | <0.001 |
|  |  | Linear | 0.694 | 0.049 | 14.220 | <0.001 |
|  | Medium-level (n=701) | Intercept | 112.555 | 0.467 | 241.269 | <0.001 |
|  |  | Linear | 0.987 | 0.053 | 18.748 | <0.001 |
|  | High-level (n=92) | Intercept | 130.384 | 1.415 | 92.125 | <0.001 |
|  |  | Linear | 1.109 | 0.139 | 7.993 | <0.001 |

# **Table S11.** Parameter estimates for polynomial terms of DBP trajectory models

| Group | Trajectory groups | Polynomial form of model | Estimate | SE | *t* | *P* |
| --- | --- | --- | --- | --- | --- | --- |
| 3 | Low-level (n=390) | Intercept | 66.388 | 0.369 | 180.120 | <0.001 |
|  |  | Linear | -0.117 | 0.048 | -2.436 | 0.015 |
|  | Medium-level (n=755) | Intercept | 73.003 | 0.384 | 190.025 | <0.001 |
|  |  | Linear | 0.091 | 0.031 | 2.904 | 0.004 |
|  | High-level (n=201) | Intercept | 81.558 | 0.521 | 156.460 | <0.001 |
|  |  | Linear | 0.371 | 0.057 | 6.560 | <0.001 |

# **Table S12.** Parameter estimates for polynomial terms of MAP trajectory models

| Group | Trajectory groups | Polynomial form of model | Estimate | SE | *t* | *P* |
| --- | --- | --- | --- | --- | --- | --- |
| 3 | Low-level (n=628) | Intercept | 79.165 | 0.283 | 279.251 | <0.001 |
|  |  | Linear | 0.207 | 0.033 | 6.196 | <0.001 |
|  | Medium-level (n=640) | Intercept | 87.668 | 0.320 | 273.541 | <0.001 |
|  |  | Linear | 0.444 | 0.039 | 11.318 | <0.001 |
|  | High-level (n=78) | Intercept | 99.499 | 1.015 | 98.006 | <0.001 |
|  |  | Linear | 1.317 | 0.295 | 4.468 | <0.001 |
|  |  | Quadratic | -0.068 | 0.0230 | -2.948 | 0.003 |

# **Table S13.** Excluding women who used antihypertensive medication during follow-up (N=67)

| Groups | SBP | DBP | | | MAP |
| --- | --- | --- | --- | --- | --- |
|  | aRR (95% CI) | | | | |
| Low-level trajectory | Ref. | | Ref. | Ref. | |
| Medium-level trajectory | 1.07 (0.91–1.18) | | 1.06(0.98–1.14) | 1.05(0.94–1.19) | |
| High-level trajectory | 1.65(1.15–2.68) | | 1.44(1.17–1.76) | 1.54(1.24–2.05) | |

**aRR:** adjusted risk ratio.

**Reference categories:** Non-APOs (for exposure); Low-level trajectory (for outcome).

**Model 1:** unadjusted; **Model 2:** adjusted for maternal age at delivery; **Model 3:** additionally adjusted for blood pressure during pregnancy, BMI during pregnancy, household income, education level, physical activity level, and alcohol consumption **based on Model 2**.

# **Figure S1.** Directed acyclic graph (DAG)

Because APOs comprise heterogeneous pregnancy complications, this DAG represents a simplified causal structure for the composite APOs exposure used in the primary analysis and does not fully capture potential causal relationships among individual APOs subtypes.
